# Professional quality of life among Spanish veterinarians

**DOI:** 10.1002/vro2.50

**Published:** 2022-11-21

**Authors:** Patricia Macía, Olatz Goñi‐Balentziaga, Oscar Vegas, Garikoitz Azkona

**Affiliations:** ^1^ Department of Basic Psychological Processes and their Development Euskal Herriko Unibertsitatea (UPV/EHU) Donostia Spain; ^2^ Department of Clinical and Health Psychology and Research Methodology Euskal Herriko Unibertsitatea (UPV/EHU) Donostia Spain

## Abstract

**Background:**

In Spain, the perceived professional quality of life among veterinarians has not been explored.

**Methods:**

Veterinarians were invited to complete an online questionnaire in which they answered the Professional Quality of Life scale, the Medical Outcomes Study Social Support Survey and the Warwick‐Edinburgh Mental Well‐Being Scale. Participants were asked whether they were receiving psychological therapy or were taking anxiolytics, hypnotics or antidepressant medication. Alcohol consumption was measured using the Alcohol Use Disorders Identification Test and nicotine dependence was assessed using the Fagerström test; participants were asked whether they took illegal drugs.

**Results:**

The study sample comprised a total of 602 veterinarians, most of whom reported average levels of compassion satisfaction, secondary stress trauma and burnout. Emotional support and mental wellbeing influenced participants’ professional quality of life. The percentage of veterinarians in psychological therapy and/or taking anxiolytics was higher than in the general population.

**Conclusions:**

A considerable number of clinical veterinarians in Spain may be suffering from work‐related stress. Our study identifies salary, emotional support and mental wellbeing as important factors that affect the professional quality of life. Interventions to improve veterinary clinicians’ professional quality of life should therefore focus on these factors.

## INTRODUCTION

In their job, veterinary clinicians can experience great joy, fulfilment and satisfaction—from caring for animals and interacting with pet owners (clients). Their work can also be distressing, particularly when they encounter barriers to caring for animals or are forced to witness their suffering. These are complex and paradoxical experiences of compassionate work in veterinary medicine.^1^

Professional Quality‐of‐Life scale (ProQOL) refers to how one feels about your work as a helper and is influenced by both the positive and the negative aspects of doing your job. On the positive side, workers may experience compassion satisfaction (CS), which refers to the pleasure that can be derived from an individual's ability to perform their job well and contribute to the work setting and the greater good of society.^2^ The factors that have been found to satisfy veterinarians are mainly linked to meaningful purpose (helping animals and clients) and self‐improvement (intellectual challenge and variety).^3^ Even so, one study has reported that veterinarians can sometimes experience low levels of CS.^4^

On the negative side, workers can experience compassion fatigue (CF), a psychological syndrome comprised of secondary traumatic stress (STS) and burnout (BO). STS is thought to occur as a result of providing care to those who have suffered or are suffering from trauma. Symptoms may include preoccupation with trauma, emotional numbing and intrusive thoughts and nightmares. BO is understood to stem from cumulative exposure to working stressors and is associated with feelings of being overwhelmed, disconnectedness and emotional exhaustion.^2^ Veterinarians in Europe, North America and Australasia have reported to have higher levels of work‐related stress, CF and BO than the general population.^4^, ^5^, ^6^, ^7^, ^8^, ^9^, ^10^, ^11^, ^12^, ^13^ However, to the best of the authors’ knowledge, no study has analysed ProQOL among veterinarians working in Spain.

Some personal and organisational factors have been found to predict ProQOL, with gender and age‐related differences, for example, being reported for CF.^5^, ^6^, ^7^, ^8^, ^12^, ^13^ The main sources of the work‐related stressors that place veterinarians at risk of CF include long working hours, low salaries, unexpected clinical outcomes, lack of social support at work and/or home, emotionally charged interactions with clients, exposure to animal suffering and euthanasia.^1^, ^9^, ^13^, ^14^, ^15^, ^16^, ^17^, ^18^, ^19^ Chronic workplace stress can lead to mood disorders and, indeed, veterinarians in different countries have reported higher levels of anxiety and depression relative to the general population.^5^, ^9^, ^11^, ^15^, ^20^, ^21^, ^22^, ^23^

Learning to cope with stress remains a critical area of veterinary clinical practice. Maladaptive (passive) coping strategies are regarded as harmful because they only enable individuals to temporarily forget stressful events and their emotional responses to them, through substance abuse, denial of emotions and isolation and avoidance strategies.^24^ Avoidant coping styles in veterinarians have been linked to increased risk of BO and suicidal ideation.^25^, ^26^ In Germany and the Unite Kingdom, practicing veterinarians/veterinary surgeons are reported to be at greater risk of alcohol or drug consumption than their non‐clinical counterparts^20^, ^27^; several studies in different countries have reported a higher‐than‐expected number of deaths from suicide among veterinarians.^9^, ^22^, ^23^, ^28^, ^29^, ^30^, ^31^ There are no statistics available for Spain on the number of suicides in the veterinary profession.

Adaptive (active) coping strategies aim to deal with the stressor in a positive manner to enable the individual to overcome the adverse event and learn from it. This may include visiting a psychiatrist or psychologist. This type of strategy is considered to be the best for coping with stress in the long‐term because it allows individuals to cope better if faced with similar situations in the future.^24^ In this sense, support from partners, family and co‐workers is always important;^16^, ^19^ as is learning to manage pet owners’ expectations.^32^, ^33^, ^34^

In the present study, our aims were to analyse perceived professional quality of life among veterinary clinicians working in Spain, to identify possible personal or professional stressors, as well as coping strategies.

## MATERIALS AND METHODS

### Participants and procedure

Participants were recruited online between 27 October 2021 and 20 January 2022, through the members’ e‐mail lists provided by different Spanish veterinary boards. The study was restricted to veterinary clinicians working in Spain. In a cover letter attached to the questionnaire, participants were informed that the survey data would be used for scientific purposes only and that they would remain completely anonymous. All participants gave their voluntary informed consent before completing the short 15‐minute online questionnaire (Google Drive platform). All procedures and informed consent protocols were approved by the Ethics Committee for Human‐Related Research (CEISH) of the University of the Basque Country (UPV/EHU); M10/2021/166.

### Instruments

The survey contained questions related to participants’ personal information, such as gender, age, marital status, the composition of their household and whether (or not) they had pets. It also contained questions related to their profession, including the type of institution in which they worked (*hospital or clinic, rural environment or other [such as zoo, animal shelter or research institute*]), their salary range, professional status (*self‐employed or employee*), professional role (*employee, team manager or director*), years working as a veterinarian, number of hours spent working directly with animals per week and the species with which they worked most often. Participants were also asked to state whether or not they were obliged to be on‐call (response options were *yes* or *no*); how much influence they felt they had over their on‐call schedule (*none, a little, some* or *a lot*); how often they had to be on‐call (*less than once a month, monthly, weekly* or *daily*); whether they wished they had more influence over their on‐call schedule (*disagree, neither disagree nor agree*, *agree*). They were asked whether they had ever euthanased an animal (*yes* or *no*); if so, how often (*less than once a month, monthly, weekly* or *daily*). Next, they were asked to respond to the statements ‘*I get to decide whether I am the one to euthanase an animal’* and ‘*the owner is present*’ by choosing one of the following options: *never, rarely, sometimes, most of the time* or *always*.

Social support was assessed using the Spanish version of the Medical Outcomes Study (MOS) Social Support Survey,^35^ which comprises four subscales: emotional support (ES), tangible support (TS), positive social interaction (PSI) and affectionate support (AS).

Subjective mental wellbeing was measured using the Spanish version of the Warwick–Edinburgh Mental Wellbeing Scale (WEMWBS ‐ 562895937).^36^

To measure participants’ perceived work‐related quality of life during the 30 days before completing the questionnaire, we used the Spanish version of the ProQOL scale^37^ adapted to animal‐care professions (by substituting the term animal for person). This scale comprises 30 items rated on a six‐point Likert‐type scale (0 = *never*; 5 = *always*) and measures two principal subscales: positive (CS) and negative (CF), with the latter being subdivided into two subscales of BO and STS.

Participants were asked whether they were receiving psychological therapy and, if so, how often they attended sessions, whether sessions were individual or group, the type of therapy they received and whether they paid for it themselves or through the social security system or private insurance. They were also asked if they were taking any prescribed anxiolytics or hypnotics and, if so, how often and what type; if they were taking prescribed antidepressants and, if so, what type. Finally, we used the alcohol use disorders identification test (AUDIT) to detect alcohol dependence^38^ and the Fagerström test to detect nicotine dependence.^39^ Participants were asked whether they took illegal drugs and, if so, how often, whether they took them alone or with others; also whether or not they had been high in the past 30 days.

### Statistical data analysis

According to the Spanish National Institute of Statistics (INE), in 2019 there were 33,752 registered veterinarians in Spain.^40^ To calculate the minimum sample required, we used the formula for quantitative variables, with a confidence level of 95%, taking into account that the scales of the different questionnaires varied between four and seven points. The minimum sample required for the study was therefore 120 subjects.

All statistical analyses were performed using the Jamovi (1.16.15; available at https://www.jamovi.org/download.html) software package, with the level of significance set to *p* < 0.05. Frequency (%) and distribution—mean ± standard deviation (SD); median/minimum–maximum—statistics were used to describe the sample. Means, SDs and homogeneity indexes were calculated for each ProQOL item. Each subscale was transformed into cut‐off scores (≤22 low, 23–41 averages and ≥42 high). Their reliability was analysed using standardised Cronbach's alpha.

The normality test (Kolmogorov–Smirnoff) revealed a non‐parametric distribution for all the scores of the variables. Subsequently, Mann–Whitney *U* tests (for variables with two categories, such as pet: yes or no) or Kruskal–Wallis one‐way analyses of variance (for variables with more than two categories, such as salary range) were conducted to analyse whether there were differences in ProQOL, MOS and WEMWBS test scores.

Rank biserial correlation and the squared‐epsilon coefficient were used to calculate effect size, with the reference values being less than 0.3 (small effect), 0.3–0.5 (moderate effect) and greater than 0.5 (large effect); and 0.01 to less than 0.06 (small effect), 0.06 to less than 0.14 (moderate effect) and less than or equal to 0.14 (large effect), respectively. Between‐group differences in psychological therapy, medication and illegal drug use were analysed using chi‐square test; if the results were significant, adjusted residuals were calculated. Cramer's *V* was used to calculate effect size, with the reference values being less than or equal to 0.2 (small effect), 0.2 to less than or equal to 0.6 (moderate effect) and greater than 0.6 (large effect).

Associations between parameters were analysed using the bivariate Spearman correlation; less than 0.09 (very small effect), 0.10–0.29 (small effect), 0.30–0.49 (moderate effect) and greater than 0.50 (large effect).

All variables, although significant, with small effects were discarded because the significance could have been due to sample size. From this first analysis, we observed that three factors (salary range, ES and mental wellbeing) can influence different subscales of ProQOL. They fulfilled the criteria of being statistically significant in the variance analysis, furthermore, they had at least a medium effect side and correlation coefficient. To determine the influence of these three variables independently, we next performed generalised linear model (GLM) analyses with three three variables predicting CS, STS and BO.

There were no missing data. Only significant differences between groups are presented in the results section; small effect sizes were not considered.

## RESULTS

### Participants’ personal and professional information

A total of 609 individuals started the survey, although four did not later agree to be included in the study and three were excluded because they were not currently working as veterinary clinicians. The sample therefore comprised a total of 602 veterinarians. Most participants were cis/trans women, aged between 23 and 65 years (40 ± 11) and almost half were in a relationship, with most living with other people and having a pet (Table 1).

**TABLE 1:** Participants’ personal information

	***n* (%)**
**Gender**	
Cis/trans women	446 (74.1%)
Cis/trans men	152 (25.2%)
Prefer not to say	4 (0.7%)
**Age range**	
23–29	116 (19.3%)
30–39	214 (35.5%)
40–49	131 (21.8%)
50–59	111 (18.4%)
60–65	30 (5.0%)
**Marital status**	
Single	272 (45.3%)
Married	202 (33.5%)
Civil partnership	81 (13.5%)
Divorced	26 (4.3%)
Separated	4 (0.6%)
Widowed	5 (0.8%)
Prefer not to say	12 (2.0%)
**Household composition**	
Live with a romantic partner	233 (38.7%)
Live with children	209 (34.7%)
Live alone	79 (13.1%)
Live with friends	51 (8.5%)
Prefer not to say	30 (5.0%)
**Pet**	
Yes	485 (80.6%)
No	117 (19.4%)

Concerning their professional category and employment status, more than half of the veterinarians in our study were employees and the vast majority worked in hospitals or clinics and earned less than 28,000 euros per year. Their experience as veterinary clinicians ranged from less than a year to 43 years (14 ± 11) and they spent 39 ± 13 hours per week working directly with animals. Fewer than 40% of participants were obliged to be on‐call and most of those were on‐call on a monthly or weekly basis. In terms of their influence over their on‐call schedule, around half had none or little influence, while the other half had some or a lot of influence. However, the majority claimed that they would like to have more influence over their on‐call schedule. Almost all participants had euthanased an animal at some point in their career, although the vast majority did so once a month or less than once a month. More than half of the participants said that it was always or mostly they who made the decision to euthanase an animal; the vast majority claimed that owners were always or mostly present during the procedure (Table 2). Most of the veterinarians participating in the study worked with dogs and cats (Table S1).

**TABLE 2:** Participants’ professional information

	***n* (%)**
**Professional category**	
Employee	355 (59.0%)
Team leader	58 (9.6%)
Manager	189 (31.4%)
**Employment status**	
Employee	398 (66.1%)
Self‐employed	204 (33.9%)
**Institution**	
Hospitals or clinics	550 (91.4%)
Rural environment	40 (6.6%)
Other	12 (2.0%)
**Salary range** (euros/year)	
12,000 to <20,000	282 (46.8%)
20,000 to <28,000	185 (30.7%)
28,000 to <36,000	68 (11.3%)
36,000 to <44,000	30 (5.0%)
44,000 to <52,000	18 (3.0%)
52,000 to <60,000	11 (1.8%)
≥60,000	8 (1.3%)
**Years worked**	
<5	158 (26.2%)
6–10	132 (21.9%)
11–20	134 (22.3%)
>20	178 (29.6%)
**Hours worked per week**	
<20	46 (7.6%)
20–40	363 (60.3%)
>40	193 (32.1%)
**On‐call**	
Yes	215 (35.7%)
No	387 (64.3%)
**On‐call frequency**	
Less than once a month	23 (10.7%)
Once a month	86 (40.0%)
Once a week	82 (38.1%)
Daily	24 (11.2%)
**Influence over own on‐call schedule**	
None	66 (30.7%)
A little	61 (28.4%)
Some	51 (23.7%)
A lot	37 (17.2%)
**Desire to have more influence over on‐call schedule**	
Disagree	17 (2.8%)
Neither disagree nor agree	24 (3.9%)
Agree	561 (93.2%)
**Euthanasia**	
Yes	596 (99.0%)
No	6 (1.0%)
**Euthanasia frequency**	
Less than once a month	204 (34.2%)
Once a month	239 (40.1%)
Once a week	145 (24.3%)
Daily	8 (1.3%)
**Euthanasia self‐determination**	
Always	227 (38.1%)
Most of the time	140 (23.5%)
Sometimes	93 (15.6%)
Rarely	64 (10.7%)
Never	72 (12.1%)
**Owners’ presence during euthanasia**	
Always	67 (11.2%)
Most of the time	349 (58.6%)
Sometimes	141 (23.7%)
Rarely	26 (4.4%)
Never	13 (2.2%)

### Social support and mental wellbeing

High levels of total social support were reported by 522 (86.7%) participants, with the rest (80/13.3%) reporting average levels. We observed the same pattern in the different MOS Social Support Survey sub‐dimensions, with the vast majority reporting high levels of ES (491/81.6%), TS (459/76.2%), PSI (518/86.0%) and AS (536/89.0%), with the rest reporting average levels in each of the MOS subscales. The WEMWBS results revealed that more than half of the veterinarians participating in our study (320/53.2%) had average levels of mental wellbeing, 228 (37.9%) high levels and 54 (9.0%) low levels (Table 3). No differences were observed in relation to personal or professional variables.

**TABLE 3:** Veterinarians’ Social Support Survey (MOS) and Warwick–Edinburgh Mental Well‐Being Scale (WEMWBS) using *t* score cut‐offs, means and standard deviations

	**Low**	**Average *n* (%)**	**High**	**Mean**	**Standard deviation**	**Median**	**Range**
Social support survey	0	80 (13.3%)	522 (86.7%)	77.3	15.60	81	23–95
Emotional support	0	111 (18.4%)	491 (81.6%)	31.8	7.51	33	10–40
Tangible support	0	143 (23.8%)	459 (76.2%)	15.7	4.35	17	4–20
Positive social interaction	0	84 (14.0%)	518 (86.0%)	16.7	3.36	18	4–20
Affectionate support	0	66 (11.0%)	536 (89.0%)	13.1	2.63	14	3–15
WEMWBS	54 (9.0%)	320 (53.2%)	228 (37.9%)	54.4	9.91	55	24–70

### Professional quality of life

The homogeneity index was above 0.30 for almost all items (Table S2). Overall, the majority of participants reported average levels of CS, STS and BO, with a few reporting low levels of CS and high levels of STS or BO (Table 4). Differences were observed between salary ranges in relation to STS (*X* ^2^_(6)_ = 42.4, *p* < 0.001, *ε* ^2^ = 0.071) and BO (*X* ^2^_(6)_ = 34.8, *p* < 0.001, *ε* ^2^ = 0.06) scores. Practitioners in the lowest salary range scored highest for STS (28.1 ± 9.3; 29/3–48) and BO (30.9 ± 6.5; 32/12–45), whereas those in the highest salary range scored lowest for those same variables (STS: 19.5 ± 8.7; 17/11–33 and BO: 23.1 ± 9.6; 22/5–36). The Spearman correlation revealed a weak negative correlation between salary ranges and STS (−0.259, *p* < 0.001) and BO (−0.228, *p* < 0.001). The ES sub‐dimension correlated negatively with BO (rho = −0.31, *p* < 0.001) and mental wellbeing correlated positively with CS (rho = 0.69, *p* < 0.001) and negatively with STS (rho = −0.45, *p* < 0.001) and BO (*r* = −0.59, *p* < 0.001). Although statistically significant, the observed effect sizes and correlations between the rest of the personal and professional variables studied were negligible or weak (Table S3).

**TABLE 4:** Veterinarians ProQOL using *t* score cut‐offs, means and standard deviations

**Professional quality of life**	**Low**	**Average *n* (%)**	**High**	**Mean**	**Standard deviation**	**Median**	**Range**
Compassion satisfaction	38 (6.3%)	443 (73.6%)	121 (20.0%)	34.4	8.12	35	4–50
Secondary trauma stress	204 (33.9%)	372 (61.8%)	26 (4.3%)	25.9	8.88	26	3–50
Burnt out	72 (12.0%)	518 (86.0%)	12 (2.0%)	29.4	6.70	30	5–45

As salary range, ES and mental wellbeing were found to be related to the ProQOL subscales, we next tested an explanatory model for predicting CS, STS and BO. The GLM analyses revealed that these three variables (salary, ES and mental wellbeing) explained 37.8% of the variance observed in CS (*F*_(9,592)_ = 41.611; *p* < 0.0001; _Adj._ *R* ^2^ = 0.378), 14.7% of the variance observed in STS (*F*_(9,592)_ = 12.505; *p* < 0.0001; _Adj._ *R* ^2^ = 0.147) and 12.5% of the variance observed in BO (*F*_(9,592)_ = 10.513; *p* < 0.0001; _Adj._ *R* ^2^ = 0.125). Only mental wellbeing had a significant effect in the three subscales (Table 5).

**TABLE 5:** General linear model analysis

	**Compassion satisfaction**			**Secondary trauma stress**			**Burnt out**		
**Variables**	***F***	***p***	***ω* ^2^**	***F***	***p***	***ω* ^2^**	***F***	***p***	***ω* ^2^**
Salary range	1.942	0.072	0.006	3.141	0.005	0.018	1.995	0.064	0.009
Emotional support	1.229	0.268	0.000	0.918	0.338	0.000	0.008	0.930	‐0.001
Warwick–Edinburgh Mental Wellbeing Scale	161.433	<0.0001	0.332	34.251	<0.0001	0.094	32.581	<0.0001	0.092

### Psychological therapy and medication

Just over one‐fifth of the participants in our study (131/21.8%) reported being in individual psychological therapy. Our results indicated no differences in relation to personal or professional variables. Most (84.7%) paid for it themselves, although in some cases the therapy was paid for by either private insurance (10.0%) or the social security system (5.3%). Participants who were in therapy reported lower CS (*U* = 20,820, *p* < 0.001, *r* = 0.321) and scored lower for mental wellbeing (*U* = 20213, *p* < 0.001, *r* = 0.341). Moreover, 38.2% went to therapy ‘less than once a month’, 19.8% had ‘monthly’ sessions, 20.6% ‘fortnightly’ sessions and 21.4% ‘weekly’ sessions. Regarding the type of treatment sought, 20.6% of participants received cognitive‐behavioural therapy (CBT), 15.3% psychoanalytic therapy, 6.1% Gestalt therapy, 3.8% solution‐focused brief therapy, 1.5% rational emotive therapy and 0.8% brief systematic therapy. The rest (51.9%) claimed not to know what type of therapy they were receiving.

When asked if they were taking anxiolytics or hypnotics, 77 participants (12.8%) said that they were, with 18.2% of these claiming to take them ‘very rarely’, 32.5% ‘once a month’, 15.5% ‘one to five times a week’ and 33.8% ‘six or more times a week’. No differences were observed in relation to personal or professional variables. Participants taking anxiolytics or hypnotics reported higher STS (*U* = 13906, *p* < 0.001, *r* = 0.304) and lower mental wellbeing (*U* = 12,219, *p* < 0.001, *r* = 0.389); those on antidepressant medication (34/5.6%) reported higher STS (*U* = 6037, *p* < 0.001, *r* = 0.357) and BO (*U* = 6441, *p* < 0.01, *r* = 0.314). Lorazepam was the most commonly prescribed anxiolytic, lormetazepam the most common hypnotic and sertraline the most common antidepressant. All prescription drugs reported by participants are listed in Table S4.

### Substance abuse

Scores on the AUDIT questionnaire revealed that 445 (74.0%) of participants were at low risk of alcoholism, 138 (22.9%) at medium risk, 15 (2.5%) at high risk and four (0.6%) were likely already alcoholics. In terms of nicotine addiction, 94 (15.6%) participants said that they smoked; 34.0% of these had very low, 21.3% low, 20.2% moderate, 22.3% high and 2.2% very high dependence. Regarding the abuse of other substances, 28 (4.7%) participants claimed to take illegal drugs. Most of these (75.0%) said they took them socially and 67.9% admitted to having been high during the last month. In terms of frequency, 35.7% took them ‘very rarely’, 21.4% ‘once a month’, 17.9% ‘one to five times a week’ and 25.0% ‘six or more times a week’. No differences were observed in relation to the rest of the variables.

## DISCUSSION

In our study, the mean levels of CS were slightly lower; while the levels of STS and BO were higher than those reported in other countries.^4^, ^6^, ^13^ Nevertheless, the percentage of participants reporting both low levels of CS and high levels of STS and BO was much lower than in these previous studies.^4^, ^6^, ^12^ Another survey reported that Spanish veterinary professionals were among the most dissatisfied in Europe and that 19% of the 380 Spanish participants reported feeling ‘completely’ burnt out.^41^ If we compare the ProQOL subscale averages found in our study with those published by others in relation to Spanish professionals working in the field of human health, both before and during the COVID‐19 pandemic, we see that veterinary clinicians scored slightly lower for CS and slightly higher for STS and BO.^37^, ^42^, ^43^, ^44^, ^45^, ^46^, ^47^, ^48^, ^49^, ^50^ Also in Spain, laboratory animal veterinarians reported higher levels of CS and lower levels of CF compared to clinical veterinarians,^51^ even though working in laboratory animal science is not perceived as socially acceptable.^52^ Low CS and BO have been found to predict intentions to leave one's current job, while BO has been found to predict intent to leave the profession.^4^ In one study, 10% of veterinary professionals in Spain stated that they wanted to leave the profession altogether.^41^

The gender profile of our participants was predominantly female, which is consistent with that observed in the profession in Spain.^41^ Our results failed to confirm the influence of many of the personal or professional factors identified previously, such as gender, age, long working hours and euthanasia. This may be due to the statistical analyses carried out as effect sizes were not calculated in many studies and were very small in ours, or because of cultural/regional differences.

The salary was the only professional variable that was related to perceived ProQOL, as reported in the United States.^13^ Financial rewards have been identified as a source of work satisfaction for veterinarians.^20^, ^53^ In Germany, for example, veterinarians belong to the upper social class.^27^ Most of our participants were employees and defined themself as women; with three out of four claimed that their salary range was in the two lowest personal income tax brackets. These data are around average for the Spanish population, but far from average for other graduate professionals, who are, on average, in the third or fourth personal income tax brackets in Spain. Furthermore, we should not overlook the gender salary gap (9.4%) that exists in Spain.^54^ The median salary earned by veterinary practitioners in our study was under 28,000 euros, whereas in Europe this figure was 39,803 euros, in 2019.^55^ Low income has been identified as a source of stress among veterinarians.^56^ These differences may be large enough to prompt many veterinarians to wonder whether it is worthwhile spending so many years studying if they are not going to be able to earn a decent living at the end of it. It is important to note that in the general population, income has been found to correlate with the quality of life and wellbeing.^57^ One report indicated that the financial impact of the pandemic on veterinary clinics in Spain was not significant; the proportion of clinic owners who said they were doing better than during the previous year has increased over the past 3 years.^41^

Another important factor determining happiness is social support,^57^ which can be defined as help from family, friends, neighbours and other members of the community.^58^ Overall, participants reported high levels of social support. In Spain, social support is viewed as a safety net that offers protection against the adverse effects of economic recessions on mental health.^59^ It is worth noting that, in our study, mental wellbeing was the only factor found to influence all three ProQOL subscales. The mean WEMWBS score in our study was seven points higher than equine veterinary surgeons during the COVID‐19 pandemic in the United Kingdom^60^; while five or six points lower than that reported among the employed population in Spain before the COVID‐19 pandemic.^36^, ^61^ Interestingly, previous studies have reported that, for people living in Spain, social support is one of the factors most closely associated with individual mental wellbeing^61^, ^62^; furthermore, there is a notable effect gradient from low to high mental wellbeing in accordance with financial difficulties.^61^

Psychological therapy, also called talking therapy, is a type of mental health treatment, alone or with medication.^63^ Participants with lower job engagement and mental wellbeing were more likely to be in psychological therapy. Perhaps due to the fact that most psychologists in Spain are in private practice, we were unable to find any current data on the number of people from the general population in psychological therapy. However, in 2014, 4.61% of the Spanish population claimed to have visited a psychologist.^64^ If we take these data into account, we see that the percentage of veterinarians in psychotherapy is five times higher than among the general population. Currently, going to psychotherapy is no longer socially frowned upon. Medication consumption records of prescription drug sales have been kept for more years. In Spain, the consumption of anxiolytics, hypnotics and antidepressants continues to grow. Consumption of anxiolytics among veterinarians was twice as high as among the general population, whereas the consumption of antidepressants was approximately 3% lower.^65^ This may indicate that the participants in our study suffered more from anxiety than from depression and that the percentage of veterinarians with anxiety is higher than among the general population, as reported in other countries.^5^, ^15^, ^17^, ^20^ Finally, alcohol is the most commonly consumed psychoactive substance, followed by tobacco and psychoactive drugs.^66^ Our results indicated that our participants were far from being considered a group with a high rate of alcohol, tobacco or illegal drug abuse, as reported in other European countries,^20^, ^27^ suggesting that Spanish veterinarians suffering from work‐related stress tend to seek professional help and social support as a coping mechanism.

The large sample was a strength of our study; however, as participants were recruited via convenience sampling, two main limitations should be noted. First, individuals with severe CF may be less likely to participate because they may be withdrawing from any additional responsibilities related to their job, meaning that we may not have collected data from this group. By conducting a face‐to‐face study, these cases could be detected. And second, veterinarians who left their jobs due to CF are also absent from the study.

Our study points to salary, ES and mental wellbeing as important factors affecting the professional quality of life. Earning a lower‐than‐expected salary may be an important factor contributing to CF and professional dissatisfaction, which in turn lead to poorer mental wellbeing. This could explain the higher rate of anxiolytic consumption and recourse to psychological therapy. Interventions designed to improve veterinary clinicians’ professional quality of life should therefore focus on these factors.

## AUTHOR CONTRIBUTIONS

Patricia Macía, Olatz Goñi‐Balentziaga and Garikoitz Azkona designed the survey and collected the data. All authors analysed the data and wrote the paper.

## CONFLICTS OF INTEREST

The authors declare they have no conflicts of interest.

## ETHICS STATEMENT

The study was conducted in accordance with the guidelines established in the Declaration of Helsinki. All procedures and informed consent protocols were approved by the Ethics Committee for Human‐Related Research (CEISH) of the University of the Basque Country (UPV/EHU); M10/2021/166.

## Supporting information

Supporting Information

## Data Availability

The study data will be made available upon reasonable request to the corresponding author.
