# Supplementary material for: Professional quality of life among Spanish veterinarians
Source: Vet Rec Open. 2022 Nov 21;9(1):e250. doi: 10.1002/vro2.50 (PMC9677367; doi:10.1002/vro2.50)
Supplement: Supplementary file 1 — Supporting Information [file VRO2-9-e250-s001.docx]

# Supporting Information

**Supporting Information Table 1**

Species by alphabetic order

|  | n (%) |
| --- | --- |
| Aquatic species | 14 (2.3%) |
| Birds | 96 (15.9%) |
| Cat *(Felis catus)* | 557 (92.5%) |
| Cow *(Bos taurus)* | 25 (4.1%) |
| Dog (*Canis lupus familiaris*) | 560 (93%) |
| Ferret *(Mustela putorius furo)* | 140 (23.3% |
| Goat *(Capra aegagrus hircus)* | 28 (4.6%) |
| Horse (*Equus ferus caballus*) | 32 (5.3%) |
| Non-human primate | 7 (1.2%) |
| Pig *(Sus scrofa)* | 25 (4.1%) |
| Rabbit *(Oryctolagus cuniculus)* | 219 (36.4%) |
| Reptiles | 66 (10.9%) |
| Rodents | 152 (25.2% |
| Sheep (*Ovis aries*) | 30 (5%) |
| Tropical species | 27 (4.5%) |
| Wild species | 13 (2.1%) |

**Supporting Information Table 2**

Mean, standard deviation and item-total correlation for each ProQOL item

| *ProQoL* | *Mean* | *Standard* | *r* |  |
| --- | --- | --- | --- | --- |
| **Compassion satisfaction (α = 0.88)** | | | | |
| 3. Estoy satisfecho por ayudar a los animales | | 4.14 | 0.96 | 0.63 |
| 6. Me siento fortalecido después de trabajar con los animales a los que he ayudado | | 3.72 | 1.14 | 0.66 |
| 12. Me gusta trabajar ayudando a los animales | | 4.35 | 0.86 | 0.60 |
| 16. Estoy satisfecho por cómo soy capaz de mantenerme al día en las técnicas y procedimientos de asistencia médica | | 3.05 | 1.22 | 0.49 |
| 18. Mi trabajo me hace sentirme satisfecho | | 3.14 | 1.19 | 0.80 |
| 20. Tengo pensamientos de satisfacción acerca de los animales a los que he ayudado y sobre cómo he podido ayudarles | | 3.67 | 1.01 | 0.65 |
| 22. Creo que puedo hace cambiar las cosas a través de mi trabajo | | 2.52 | 1.34 | 0.48 |
| 24. Planteo continuar con mi trabajo por muchos años | | 2.94 | 1.53 | 0.58 |
| 27. Considero que soy un buen profesional | | 3.67 | 0.95 | 0.47 |
| 30. Estoy feliz por haber elegido hacer este trabajo | | 3.23 | 1.45 | 0.73 |
| **Burnout (α = 0.64)** | | | | |
| 1. Soy feliz (R) | | 2.82 | 1.05 | 0.44 |
| 4. Me siento vinculado a otras personas, con ocasión de mi trabajo (R) | | 2.49 | 1.10 | 0.20 |
| 8. Pierdo el sueño por las experiencias traumáticas de los animales a los que he ayudado | | 3.00 | 1.46 | 0.37 |
| 10. Me siento "atrapado" por mi trabajo | | 3.45 | 1.40 | 0.52 |
| 15. Tengo creencias (religiosas, espirituales u otras) que me apoyan en mi trabajo profesional (R) | | 1.63 | 2.01 | 0.06 |
| 17. Soy la persona que siempre he querido ser (R) | | 2.85 | 1.23 | 0.34 |
| 19. Por causa de mi trabajo me siento agotado | | 3.71 | 1.18 | 0.59 |
| 21. Me siento abrumado por la cantidad y tipo de trabajo que tengo que afrontar | | 3.45 | 1.22 | 0.53 |
| 26. Me siento “estancado” (sin saber qué hacer) por cóm funciona el sistema sanitario | | 3.08 | 1.50 | 0.52 |
| 29. Soy una persona demasiado sensible (R) | | 2.88 | 1.40 | -0.17 |
| **Secondary Traumatic Stress (α = 0.85)** | | | | |
| 2. Estoy preocupado/a por un o más animales a los que he ayudado o ayudo | | 4.01 | 0.95 | 0.43 |
| 5. Me sobresaltan los sonidos inesperados | | 2.52 | 1.48 | 0.40 |
| 7. Encuentro difícil separar mi vida personal de mi vida profesional | | 3.63 | 1.33 | 0.44 |
| 9. Creo que he sido afectado negativamente por las experiencias traumáticas de aquellos a quienes he ayudado | | 2.77 | 1.42 | 0.71 |
| 11. Debido a mi profesión tengo la sensación de estar al límite en varias cosas | | 3.47 | 1.34 | 0.63 |
| 13. Me siento deprimido como resultado de mi trabajo | | 2.43 | 1.35 | 0.68 |
| 14. Me siento como si fuera yo el que experimenta el trauma de alguno al que he ayudado | | 2.01 | 1.40 | 0.68 |
| 23. Evito ciertas actividades o situaciones porque me recuerdan a las experiencias espantosas de los animales a los que he ayudado | | 1.48 | 1.40 | 0.57 |
| 25. Como resultado de mi trabajo profesional, tengo pensamientos molestos, repentinos, indeseados. | | 2.32 | 1.51 | 0.71 |
| 28. No puedo recordar determinados acontecimientos relacionadas | | 1.26 | 1.23 | 0.34 |

*Notes* (R) reverse items; *r* = corrected item-total correlation.

**Supporting Information Table 3**

Analysis of variance and correlation related to professional quality of life

|  | **Analysis of variance** | **Size effect** | **Correlation** |
| --- | --- | --- | --- |
| **Compassion Satisfaction** | | | |
| Age | X^2^_(4)_ = 13.9** | ε^2^ = 0.02 | Nc no correlation |
| Pet | U = 23097* | r = 0.1574 | rho = -0.108* |
| Salary | X^2^_(6)_ = 25.4*** | ε^2^ = 0.042 | rho = 0.164** |
| Worked years | X^2^_(4)_ = 13.9** | ε^2^ = 0.02 | rho = 0.086* |
| On-call timetable influence | Ns not significant | ns | rho = 0.17* |
| Desired influence on on-call timetable | ns | ns | rho = -0.168* |
| Euthanasia frequency | X^2^_(3)_ = 15.9** | ε^2^ = 0.027 | rho = -0.150*** |
| Euthanasia decision | X^2^_(4)_ = 24.9*** | ε^2^ = 0.0421 | rho = 0.179*** |
| **Secondary Trauma Stress** | | | |
| Gender | X^2^_(2)_ = 23.08*** | ε^2^ = 0.038 | rho = -0.196*** |
| Age | X^2^_(4)_ = 26.5** | ε^2^ = 0.04 | rho = -0.186*** |
| Salary | X^2^_(6)_ = 42.4*** | ε^2^ = 0.071 | rho = -0.259*** |
| Institution | X^2^_(2)_ = 13.96*** | ε^2^ = 0.023 | rho = -0.152*** |
| Worked years | X^2^_(4)_ = 26.5*** | ε^2^ = 0.04 | rho = -0.191*** |
| Worked hours/week | X^2^_(2)_ = 16.61*** | ε^2^ = 0.03 | rho = 0.157*** |
| Desired influence on on-call timetable | ns | ns | rho = 0.138* |
| Euthanasia frequency | X^2^_(3)_ = 14.6* | ε^2^ = 0.0247 | rho = 0.155*** |
| Euthanasia decision | X^2^_(4)_ = 18.8*** | ε^2^ = 0.0318 | rho = -0.161*** |
| **Burn Out** | | | |
| Age | X^2^_(4)_ = 23.7** | ε^2^ = 0.04 | rho = -0.141*** |
| Institution | X^2^_(6)_ = 16.64*** | ε^2^ = 0.028 | rho = -0.165*** |
| Worked years | X^2^_(4)_ = 23.7*** | ε^2^ = 0.04 | rho = -0.174*** |
| Worked hours/week | X^2^_(2)_ = 26.77*** | ε^2^ = 0.05 | rho = 0.210*** |
| Desired influence on on-call timetable | ns | ns | rho = 0.154* |
| Euthanasia frequency | X^2^_(3)_ = 19.2*** | ε^2^ = 0.0324 | rho = 0.175*** |
| Euthanasia decision | X^2^_(4)_ = 16.8** | ε^2^ = 0.0284 | rho = -0.157*** |
| *Notes* * p < 0.05, ** p < 0.001, *** p < 0.001.  Subscripts = degrees of freedom | | | |

**Supporting Information Table 4**

Anxiolytic, hypnotic and antidepressant medication reported by participants

|  | Number of participants |
| --- | --- |
| **Anxiolytics** | |
| Alprazolam | 13 |
| Bromazepam | 9 |
| Clobazam | 2 |
| Clorazepate dipotassium | 1 |
| Diazepam | 24 |
| Lorazepam | 33 |
| **Hypnotics** | |
| Loprazolam | 2 |
| Lormetazepam | 4 |
| Midazolam | 2 |
| Zolpidem | 1 |
| **Antidepressants** | |
| Amitriptyline | 2 |
| Citalopram | 1 |
| Desvenlafaxine | 2 |
| Duloxetine | 2 |
| Escitalopram | 6 |
| Fluoxetine | 6 |
| Mirtazapine | 4 |
| Paroxetine | 3 |
| Sertraline | 9 |
| Trazodone | 1 |
| Venlafaxine | 2 |
| Vortioxetine | 2 |
